# Enhancing Chinese students’ academic engagement: the effect of teacher support and teacher–student rapport

**DOI:** 10.3389/fpsyg.2023.1188507

**Published:** 2023-06-15

**Authors:** Xiaoquan Pan, Yuanyuan Yao

**Affiliations:** ^1^Xingzhi College, Zhejiang Normal University, Jinhua, China; ^2^College of Foreign Languages, Zhejiang Normal University, Jinhua, China

**Keywords:** academic engagement, teacher support, teacher–student rapport, Chinese undergraduate students, empirical investigation

## Abstract

Academic engagement plays an undeniable role in students’ leaning outcome. Therefore, identifying the influential antecedents of promoting students’ academic engagement is extremely crucial. Despite previous empirical studies have delved into the part played by several student-related and teacher-related factors in triggering Chinese students’ academic engagement, the exploration on the roles of teacher support and teacher–student rapport is still scant. Thus, this study attempts to concentrate on the influence of teacher support and teacher–student rapport on undergraduate students’ academic engagement in China. Three scales of the questionnaire—one each for teacher’s support, student-teacher rapport, and the level of academic engagement—were completed by a total of 298 undergraduate students. Spearman Rho test was adopted to detect the correlations between the variables. Following that, multiple regression analysis was used to estimate the predictive power of the dependent variables. The result found that teacher support and teacher–student rapport exert a tremendous influence on boosting Chinese students’ academic engagement. The leading implications and future directions are also presented.

## 1. Introduction

Students’ academic engagement is considered to be a crucial component to positive academic behaviors (Peng, 2021), social, or extracurricular activities (Snijders et al., 2020) in the educational context (Jiang and Zhang, 2021), especially for teenagers who are confronted with emerging challenges and academic needs (Geng et al., 2020). Engagement is acclaimed as a state of greater focus and active participation in which involvement is not only cognitive, but also social, behavioral, and emotional (Philp and Duchesne, 2016). Students who are actively engaged in their learning experiences are found to show energy, flexibility, and positive emotions, which raises their likelihood of academic performance, positive adolescent development results, and positive instructor attention (Skinner et al., 2008; Oga-Baldwin and Nakata, 2017; Quin, 2017; Wang, 2022). More specifically, high levels of learner engagement have been evidenced to be correlated with an array of optimistic academic performance, including enhanced academic tenacity, dedication, academic desires, fulfillment, increased mental health, low levels of student boredom, alienation, degree completion, dropout rates, and reduced high-risk behaviors (Snijders et al., 2020; Hiver et al., 2021). Nevertheless, the predictors of students’ academic engagement have remained unknown (Wang, 2022).

In light of this, extensive empirical studies investigating student engagement and its potential antecedents have been conducted. As an instance, in examining the effects of second language learning (L2) enjoyment and academic motivation on Chinese students’ participation in English as a foreign language (EFL) classes, Wang (2022) discovered that both factors exerted a significant influence on Chinese students’ academic engagement. More specifically, the more academic engagement students demonstrate, the higher the level of L2 enjoyment and academic enthusiasm they possess. In addition, existing studies of teachers’ instructional roles have focused on how elements associated to teachers can help enhance students’ active engagement in their academic study, including teacher caring behavior and teacher praise (Peng, 2021; Sun, 2021; Derakhshan et al., 2022), teacher stroke (Yuan, 2022), and teachers’ teaching style (Jiang and Zhang, 2021). However, according to Sun and Shi (2022), previous research have not definitely highlighted the influencing mechanisms of teacher support and teacher–student rapport as two significant teacher-related factors on academic engagement. That is to say, it has remained challenging to figure out the amount to which teacher support and student-teacher rapport can foster Chinese students’ academic engagement. The current research endeavors to investigate the impact of teacher support and teacher–student rapport on Chinese students’ academic engagement so as to address this need.

Teacher support, as a potential driver of academic engagement, pertains to “the extent to which learners believe their teachers value and seek to establish personal relationships with them” (Chong et al., 2018, p. 3). The extant literature highlighted the influence of teachers’ behavior supports on students’ cognitive, affective and social learning behaviors (Farmer et al., 2011) as well as students’ learning beliefs and approaches (Davis, 2003). An empirical study conducted by Alrashidi et al. (2016) indicated that one strategy to strengthen and improve students’ proactive engagement in academic and school-related activities is through teacher support. According to Liu et al. (2021), teacher support may be incredibly crucial to middle school students and has an enormous impact on students’ motivational beliefs. Hence, teacher assistance is vital in elevating middle school students’ creative self-efficacy in academic settings. Similarly, Sun and Shi (2022) advanced that Chinese students’ affective learning can be strongly impacted by their teachers, therefore EFL learners who get consistent help and support are more likely to achieve good learning results.

Another teacher-related factor that may influence students’ academic engagement is teacher–student rapport. In terms of educational psychological context, it is deemed as the trusting relationship which can connect teachers and students in a certain emotional atmosphere and experience and realize the transmission and communication of emotional information in numerous positive interpersonal interactions (Derakhshan et al., 2022). As stated by Skinner et al. (2008), academic engagement, concentration in the classroom, love of the school, and expectations for academic success are all strongly correlated with the quality of student-teacher connections in the form of loving, encouraging alliances. Besides, Quin (2017) elucidated that teacher–student relationships both directly and indirectly influenced multiple indicators of students’ active learning engagement. It’s conceivable that good teacher–student relationships will stimulate students’ desire to learn. Similarly, Xie and Derakhshan (2021) also delineated that an array of desirable students’ academic outcomes, such as active learning engagement behaviors, are strongly facilitated by positive teacher–student interpersonal relationships.

Acknowledging the utmost important of teacher support and student-teacher relationship in pedagogical dimensions (Sadoughi and Hejazi, 2021; Shen and Guo, 2022; Sun and Shi, 2022), a handful of investigations have been made to achieve their desired outcomes (e.g., Pitzer and Skinner, 2017; Lei et al., 2018; Liu et al., 2021; Affuso et al., 2022; Sun and Shi, 2022). Yet, there has not been much focus on how these constructs can enhance students’ academic engagement. Moreover, grounded on the existing literature, we assume that few empirical studies have simultaneously conducted on these two teacher-related factors to identify their effects on enhancing Chinese students’ academic engagement. By inspecting the importance of teacher support and teacher–student rapport in promoting Chinese students’ academic engagement, this investigation attempts to eliminate the aforementioned gaps.

## 2. Literature review

### 2.1. Teacher support

Teachers exert a significant influence on students’ cognitive, affective, and social learning behaviors, which considerably affects the quality of their learning experiences (Pan and Chen, 2021). Consequently, in the classroom, teachers are pivotal in the dissemination of knowledge, instruction, and development of students’ academic performance (Chong et al., 2018). As shown by self-determination theory (Ryan and Deci, 2020), teacher support signifies that teachers proactively address students’ three core psychological needs of autonomy, competence, and relatedness. As reported in previous studies (Lei et al., 2018; Liu et al., 2021; Sadoughi and Hejazi, 2022; Wu and Yang, 2022), teacher support, as a multidimensional construct, can be classified as academic support, emotional support, and competence support, appraisal support, and instrumental support etc. Academic support refers to students’ belief that their instructors care about what they have learned, how much they have learned, and how they can help them learn (Liu et al., 2021; An et al., 2022; Wu and Yang, 2022). Emotional support is marked by compassion, kindness, respect, inspiration, and care (Utvær et al., 2022). Teachers’ remarks, judgments, and evaluations of students’ performance, together with their recommendations and suggestions, are referred to as appraisal support (Sadoughi and Hejazi, 2022). Instrumental support involves teachers’ tangible resource in terms of skills, services, time and money (Lei et al., 2018; Sadoughi and Hejazi, 2022).

### 2.2. Teacher–student rapport

In terms of instructional context, Rapport, generally speaking, was conceptualized as “an overall feeling between two people encompassing a mutual, trusting, and prosocial bond” (Frisby and Martin, 2010, p. 147). Further, it was described as an interrelationship formed between teachers and students in the process of education and teaching, characterized by satisfaction, connectedness, respect, and interpersonal trust (Delos Reyes and Torio, 2021). Friendship, mutual respect and appreciation, caring and delight can all be signs of rapport (Delos Reyes and Torio, 2021; Meng, 2021). According to Zhou (2021), since teacher–student rapport is a strong connection between educators and learners that allows them to collaborate in classroom settings, any emphasis on education cannot be detached from spotlighting it (Yuan, 2022). As Aldrup et al. (2018) addressed, teacher–student relationships constitute essential components of educational environments; however, even for many experienced teachers, the task of building and keeping an encouraging interpersonal relationship is extremely challenging. Considering the significance of teacher–student relationships in the educational realm, a range of researchers have proposed ways in which teachers can build a good and strong rapport with learners. According to Yang (2021), when students receive positive feedback, it appears to increase their enthusiasm for learning and makes learning contexts much more pleasurable, leading in a willing to interact between teachers and students. In a similar vein, Santana (2019) proposed seven methods which are conduce to build positive rapport with learners, such as understanding the students, encouraging the students and caring, communicating effectively, being approachable, being respectful and respected, being authentic, and using humor.

### 2.3. Academic engagement

Students’ academic engagement is considered as “the time and energy students devote to educationally sound activities inside and outside of the classroom, and the policies and practices that institutions use to induce students to take part in these activities” (Kuh, 2003, p. 25). Further, Skinner et al. (2009) proposed that engagement pertains to “the quality of a student’s connection or involvement with the endeavor of schooling and hence with the people, activities, goals, values, and place that compose it” (p. 494). When it comes to the language learning context, Hiver et al. (2021) defined academic engagement as “how actively involved a student is in a learning task and the extent to which that physical and mental activity is goal-directed and purpose-driven” (p. 3). Finally, taking all above definitions in consideration, academic engagement is conceptualized by Alrashidi et al. (2016) as “a positive and proactive term that captures students’ quality of participation, investment, commitment, and identification with school and school-related activities to enhance students’ performance” (p. 42). Despite the growing attention in student academic engagement (Brint et al., 2008; Skinner et al., 2008; Peng, 2021; Wang, 2022), the term “academic engagement” is interpreted in an array of ways and is occasionally employed interchangeably with terms like “school engagement,” “student engagement in school,” “engagement in class,” and “engagement in schoolwork” (Schaufeli et al., 2002; Fredricks et al., 2004; Darr, 2012;Alrashidi et al., 2016; Peng, 2021). Likewise, the numbers and sorts of elements that make up this structure have been the focus of a protracted argument (Alrashidi et al., 2016; Peng, 2021). For example, Fredricks et al. (2004) categorized student academic engagement into three components (i.e., behavioral, emotional, and cognitive), whereas Reeve and Tseng (2011) divided this construct into four main dimensions (i.e., behavioral, emotional, cognitive, and agentic) (Oga-Baldwin, 2019). According to Pedler et al. (2020), student engagement is generally viewed as a ductile, multidimensional construct that incorporates the three aspects of behavioral, emotional, and cognitive engagement. The term “behavioral engagement” refers to the visible academic performance and wanting to share tasks and activities that are assessed through observable educational acts such as: student desirable outcome, greater involvement, endeavor to concentrate on practices, willingness to participate in class discussions, desire to contribute in educational activities (Sun, 2021). Students’ emotional states throughout educational process (such as curiosity, delight, passion, boredom, happiness, sadness, and anxiety) alongside their sense of identification and relatedness to counterparts, educators, and the school are all considered to be part of emotional engagement (Darr, 2012; Lawson and Lawson, 2013; Geng et al., 2020). Cognitive engagement entails the way learners proactively engage about the subject they are learning by elucidating meanings, drawing connections, addressing problems, memorizing concepts, and responding to questions (Oga-Baldwin and Nakata, 2017). According to Weyns et al. (2018) and Skinner et al. (2008), teacher support and teacher–student rapport were demonstrated to foretell academic engagement as two external factors cultivating optimistic child development at school. However, few studies have considered both teacher support and teacher–student rapport as factors that enhance students’ academic engagement. Additionally, grounded on the existing literature, few investigations have assessed the simultaneous role of teacher support and teacher–student rapport. The present study intended to close this gap by exploring the effect of teacher support and teacher–student rapport on academic engagement. Informed by the above discussed, two overarching research questions were generated:

(1) What are the relations between academic engagement, teacher support, and teacher–student rapport among Chinese students?
(2) Do teacher support and teacher–student rapport significantly enhance Chinese students’ academic engagement?

## 3. Methodology

### 3.1. Participants

A total of 305 undergraduate students in a comprehensive university in China, aged from 18 to 22 (average age = 20, SD = 1.52), were enrolled to participate in the study using the convenience sampling strategy. Nevertheless, owing to incomplete questionnaires, 7 students’ data were deleted. Thus, the final sample was 298, consisting of 104 (34.9%) males and 194 (65.1%) females. Participants varied regarding grade, ranging from freshmen to seniors. Before collecting the data, participants were all informed of the research purpose and of their voluntary participation. Meanwhile, they received reassurances that their answers and personal data would be treated in strict confidence.

### 3.2. Instruments

#### 3.2.1. Teacher support

The questionnaire scale of teacher support, adapted from Babad (1990) and Ouyang (2005), served to gauge how an instructor is perceived by Chinese undergraduates as being supportive. An exploratory factor analysis (EFA) was conducted to remove garbage items and thereby this scale of teacher support totally involved 18 items of three dimensions: academic support (6 items, e.g., “When we are unable to answer a question, the instructor frequently repeats the explanation”), emotional support (6 items, e.g., “The instructor usually supports and cares about our academic performance”), and competence support (6 items, e.g., “My teacher frequently suggests that I take part in various competitions”). Each item was assessed on a six-point Likert scale ranging from 1 (strongly disagree) to 6 (strongly agree). Higher scores indicated better perceptions of teacher support. The Cronbach’s *α* value of teacher support scale was 0.95, indicating good reliability.

#### 3.2.2. Teacher–student rapport

The Wilson et al. (2010)‘s “Professor-Student Rapport Scale (P-SRS)” was modified to measure students’ perceptions on the quality of their relations with teachers. The scale encompasses 6 items, each of which is scored on a six-point Likert scale ranging from 1 (strongly disagree) to 6 (strongly agree). One sample item is “The teacher encourages students’ questions and comments.” In this study, the Cronbach’s *α* value of this scale was 0.93, showing good reliability.

#### 3.2.3. Academic engagement

Reeve and Tseng’s (2011) Academic Engagement Scale and Wang et al. (2016) The Math and Science Engagement Scales were both modified to measure academic engagement. Some items were changed and produced in response to the realistic situation. EFA was conducted to spot and remove unrelated items, thus remaining 18 items. This six-point Likert scale involves three sub-scales: behavioral engagement (6 items, e.g., “I actively answer the questions asked by the teacher in class”), emotional engagement (6 items, e.g., “I eagerly await English class”), and cognitive engagement (6 items, e.g., “No matter how difficult the learning content is, I will not give up and try to understand them”). With the Cronbach’s *α* value of 0.96, a good reliability of this scale was found in the present study.

### 3.3. Procedure and data analysis

To begin with, the above-mentioned three scales were distributed to the participants on the spot during lesson intervals and collected immediately after completion. Moreover, all respondents received instructions on how to answer to the survey questions, which heightened the accuracy and credibility of responses. After completing the procedure of data collection, the respondents’ answers were further reviewed to ensure the reliability of the collected data. Afterward, the SPSS 21.0 was adopted to conduct Spearman correlation analyses to assess the relationships between teacher support, teacher–student rapport, and academic engagement. At last, to ascertain how much of the variance in Chinese students’ academic engagement may be ascribed to teacher support and teacher–student rapport, multiple regression analyses were carried out.

## 4. Results

First of all, the normality of the gathered data was verified utilizing Kolmogorov–Smirnov test. The results are shown in Table 1.

**Table 1:** Results of Kolmogorov–Smirnov test.

Kolmogorov–Smirnov			
	Statistic	df	Sig.
Teacher support	0.196	298	0.000
Teacher–student rapport	0.240	298	0.000
Academic engagement	0.137	298	0.000

As depicted in Table 1, the data distribution of all of the three questionnaires showed abnormal. Therefore, non-parametric tests were needed to calculate possible associations between variables. The relations between teacher support, teacher–student rapport, and academic engagement were examined using the Spearman correlation test. Table 2 showed the results of the Spearman correlation test.

**Table 2:** Results of Spearman correlation test.

Constructs	Teacher support	Teacher–student rapport	Academic engagement
Teacher support	(0.95)		
Teacher–student rapport	0.815^**^	(0.93)	
Academic engagement	0.693^**^	0.609^**^	(0.96)

*N* = 298. ^**^Correlation is significant at the 0.01 level (2-tailed). Reliabilities (Cronbach *α*) are shown on the diagonal in parentheses.

As Table 2 delineated, there is a strong and largely favorable association between teacher support and teacher–student rapport (*r* = 0.815, *p* < 0.001). The results of Spearman correlation indicated a significant relationship (*r* = 0.693, *p* < 0.001) between academic engagement and teacher support. Academic engagement was also very closely related to teacher–student relationship (*r* = 0.609, *p* < 0.001). That is to say, the higher the level of teacher support and teacher–student rapport predicts the greater degree of academic engagement. Afterwards, to investigate the role of teacher support and teacher–student rapport in influencing academic engagement in Chinese students, multiple regression analysis was implemented. Table 3 displayed the results of the regression analysis.

**Table 3:** Model summary for teacher support, teacher–student rapport, and academic engagement.

Model	*R*	*R* square	Adjusted *R* square	Std. error of the estimate	Durbin–Watson
1	0.639^a^	0.408	0.404	7.01	1.578

a Predictors: teacher support, teacher–student rapport.

Based on Table 3, teacher support and student–teacher rapport account for 40.8% of the variation in Chinese students’ academic engagement. Beta coefficient table was subsequently conducted to discern the results of independent variables (teacher support and teacher–student rapport) contributing to enhanced academic engagement. From the beta column (Table 4), the beta coefficient of teacher support was 0.171 with a statistical significance (*p* < 0.05), indicating the positive stimulating effect of teacher–student rapport on students’ academic engagement. Furthermore, the beta coefficient of teacher support was 0.485 with a statistical significance (*p* < 0.001), which contributed the most to the improvement of Chinese students’ academic engagement. In other words, it was discovered that teacher support was a great indicator of Chinese students’ academic engagement.

**Table 4:** The coefficients for teacher support, teacher–student rapport, and academic engagement.

Model		Unstandardized coefficients		Standardized coefficients	*t*	Sig.
		*B*	Std. error	Beta		
1	(Constant)	0.085	0.347		0.246	0.806
	Teacher support	0.679	0.126	0.485	5.396	0.000
	Teacher–student rapport	0.222	0.117	0.171	1.903	0.038

Dependent variable: academic engagement.

## 5. Discussion

The primary intention of this study was to shed light on the degree to which Chinese students’ academic engagement is correlated with teacher support and teacher–student rapport. The Spearman correlation test unveiled, firstly, a straightforward and powerful correlation between teacher support and academic engagement, and secondly, a positive association of teacher–student rapport and academic engagement. On the obvious association between academic engagement and support from teachers, this finding is in line with the study of Sadoughi and Hejazi (2021), which identified teacher support could have a direct and positive impact on EFL learners’ academic engagement. It is noteworthy that this result is also consistent with that of Zhou et al. (2022), who found that all three dimensions of teacher support were favorably correlated with students’ learning engagement. Just as the theory of self-determination concentrating largely on how environments influence people’s psychological needs (Jeno et al., 2019), as an important part of social support, teacher support helps cultivate students’ autonomy and encourages students to explore independently, so that they can actively face challenges in learning. In addition, teacher support promotes students’ internal motivation and enhances students’ concentration in the learning process, thus giving rise to a higher level of academic engagement. Regarding the favorable correlation between teacher–student rapport and academic engagement, this lends support to Engels et al. (2021), who stated warm and intimate teacher–student relationships contribute to adolescents’ school engagement. This also accords with the previous study from Zhou (2021), who summarized that rapport between teachers and learners is desirable to arouse students’ academic engagement. This result also further reinforces what Xie and Derakhshan (2021) have put emphasis on the significance of teacher–student rapport, namely, when an effective teacher–student rapport is established, positive student outcomes, including L2 engagement, are on the horizon.

Besides, this study attempted to ascertain the worth of teacher support and teacher–student rapport in fostering students’ academic engagement. That is to say, this study was to investigate how much teacher support and teacher–student rapport exert influence on the enhancement of students’ academic engagement. As illustrated by the results of the regression analysis, teacher support has proven to be a robust predictor of Chinese students’ academic engagement. Chinese students’ academic engagement is positively affected by teacher support, as students who are capable of receiving adequate and prompt assistance from their teachers are prone to enjoy learning and are inclined to put forth more time and effort into it (Zhao and Yang, 2022). Such an explanation aligns with An et al. (2022) who uncovered a strikingly remarkable correlation between teacher support and academic engagement in a technology-related educational setting. To be specific, students will get involved in learning deeper if teachers provide them with more emotional and behavioral support in contexts that contain technology. This finding also echoes the idea of Ruzek et al. (2016), who posited that teacher behaviors, such as offering timely academic and emotionally caring feedback to students, complimenting and respecting students, are notably linked to students’ academic engagement, which contributed to an increase in students’ desire to be engaged in learning activities. As with teacher support, teacher–student rapport is a powerful antecedent to students’ engagement as well. This is in accord with the finding of Cooper (2014) study, who stated that active relationships between teachers and students can be a crucial resource for underpinning students’ academic endeavors as well as stimulating students to become deeper engaged in their studies.

## 6. Conclusion

The primary aim of the current study was to figure out how much academic engagement among Chinese students is influenced by teacher support and teacher–student rapport. Multiple regression analysis and Spearman Rho correlation analysis indicated that teacher support and teacher–student rapport could dramatically boost Chinese students’ academic engagement. Therefore, students are more probable to be involved in a range of learning tasks and put efforts into them if they get enough support and encouragement from teachers and develop friendly relationships with them. This result does seem to be impressively profound for educators and teachers, especially those who are now engaged in teaching in EFL environments. That is because notwithstanding the indispensable role of teacher–student rapport in any learning setting, the task of establishing and upholding a positive interpersonal relationship can be a daunting challenge, even for many well-prepared teachers. Given the findings of this investigation, teachers are encouraged to be supportive of their students as much as possible and to develop a friendly and harmonious relationship with them so that they can be more engaged in the learning process. Considering the contribution of teacher–student rapport in increasing students’ academic engagement, teachers can undertake diverse initiatives, including giving timely positive feedback to students, listening carefully and pondering on learners’ voices, so as to get the students to be engaged in the classroom. Given that teacher support significantly improves students’ academic engagement, teachers entail to provide student with constant support and assistance.

In spite of research implications found, several limitations exist in this study. Firstly, the cross-sectional quantitative approach was applied merely to conduct this exploration, which may give rise to a potential bias. Upcoming studies are suggested to utilize longitudinal method to obtain more inclusive results. Secondly, in this study, the research sample is comparatively small. Hence, further studies can adopt large-scaled survey to further assess the results. Thirdly, it needs to be highlighted that contextual factors including gender, age, and educational background were neglected and ought to be inspected in ongoing studies.

## Data availability statement

The original contributions presented in the study are included in the article/supplementary material, further inquiries can be directed to the corresponding author.

## Ethics statement

Ethical review and approval was not required for the study on human participants in accordance with the local legislation and institutional requirements. Written informed consent for participation was not required for this study in accordance with the national legislation and the institutional requirements.

## Author contributions

XP conceived, designed, and executed the study, collected the data, wrote the manuscript, and revised the final version of the manuscript. YY analyzed the data and participated in writing and revising the manuscript. All authors contributed to the article and approved the submitted version.

## Funding

This work was supported by the Research Project of the 2021 Teaching Reform of Xingzhi College, Zhejiang Normal University (Grant no. ZC303921073).

## Conflict of interest

The authors declare that the research was conducted in the absence of any commercial or financial relationships that could be construed as a potential conflict of interest.

## Publisher’s note

All claims expressed in this article are solely those of the authors and do not necessarily represent those of their affiliated organizations, or those of the publisher, the editors and the reviewers. Any product that may be evaluated in this article, or claim that may be made by its manufacturer, is not guaranteed or endorsed by the publisher.
